# ﻿Two new species of *Bamazomus* Harvey, 1992 from southern China (Schizomida, Hubbardiidae)

**DOI:** 10.3897/zookeys.1204.121754

**Published:** 2024-06-12

**Authors:** Tao Zheng, Jiaxian Gong, Feng Zhang

**Affiliations:** 1 Key Laboratory of Zoological Systematics and Application, College of Life Sciences, Hebei University, Baoding, Hebei 071002, China; 2 Hebei Basic Science Center for Biotic Interaction, Hebei University, Baoding, Hebei 071002, China; 3 College of Bee Science and Biomedicine, Fujian Agriculture and Forestry University, Fuzhou, Fujian 350002, China

**Keywords:** Asia, morphology, short-tailed whipscorpions, taxonomy

## Abstract

Two new schizomid species belonging to *Bamazomus* Harvey, 1992 are described from China: *B.shanghang***sp. nov.** (♂♀) from Fujian Province and *B.songi***sp. nov.** (♂♀) from Guangdong Province. In addition to their descriptions, illustrations and diagnoses, a distribution map is provided. These are first *Bamazomus* species from the mainland China and the northernmost in continental Asia.

## ﻿Introduction

Schizomida is an understudied arachnid order mainly distributed in tropical and subtropical areas. The order is a homogeneous group of 372 species unequally divided between Hubbardiidae Cook, 1899 and Protoschizomidae Rowland, 1975. In addition, there are 14 fossil species currently placed in Calcitronidae Petrunkevitch, 1945 (two species) and Hubbardiidae (12 species). Hubbardiidae, which is distributed worldwide, is the largest schizomid family with 356 species placed in 69 genera; among them there are three genera and four species recorded from China (WSC 2024).

The genus *Bamazomus* Harvey, 1992 was erected to receive *B.bamaga* Harvey, 1992. The taxonomically diagnostic characters are female genitalia with a gonopod and numerous lobes and a dorso-ventrally flattened male flagellum. Currently, *Bamazomus* comprises 11 species distributed from Madagascar to Australia, except for one widespread, introduced species, *B.siamensis* (Hansen, 1905), which has been recorded from Hong Kong (China), Hawaii (USA), Ryukyu Islands (Japan), and Bangkok (Thailand) (Cokendolpher and Reddell 1986; Cokendolpher 1988; WSC 2024). It is the only *Bamazomus* species known from China and from continental Asia (Bangkok).

Extensive collecting in 2022 and 2023 from southern China helped us gain a deep understanding of the natural habitat of schizomids and allowed us to obtain additional specimens of this group. In this paper, two new *Bamazomus* species, *B.shanghang* sp. nov. and *B.songi* sp. nov. are described from China.

## ﻿Materials and methods

Specimens are deposited in the Museum of Hebei University (**MHBU**), Baoding, China. All measurements in the text are given in millimetres, and the total length excludes the flagellum. The spermathecae were removed and cleared in a pancreatin solution (Álvarez-Padilla and Hormiga 2007) and then transferred to 75% ethanol for drawing. All specimens are preserved in 75% alcohol. Photographs were taken using the Leica M205A stereomicroscope equipped with a DFC 550 CCD camera and edited with Adobe Photoshop CC 2019. Morphological terminology for legs and palps follows Reddell and Cokendolpher (1995), cheliceral setae nomenclature follows Lawrence (1969) as modified by Villarreal et al. (2016), flagellar setae terminology follows Cokendolpher and Reddell (1992) as modified by Harvey (1992) and Monjaraz-Ruedas et al. (2016), palpal setae terminology follows Monjaraz-Ruedas et al. (2017), spermathecae nomenclature follows Moreno-Gonzalez et al. (2014), and opisthosomal setae nomenclature follows Villarreal et al. (2016).

Abbreviations: **AB** anterior branch of chitinized arch, **AT** accessory tooth of movable finger, **Dm** dorso-median setae of abdomen and flagellum, **Dl** dorso-lateral setae of the abdomen and flagellum, **Fe** femur ectally, **Fed** femur dorso-ectally, **Fev** femur ventro-ectally, **Fm** femur mesally, **Fmd** femur dorso-mesally, **Fmv** femur ventro-mesally, **G** setal group numbers of chelicerae, **GT** guard tooth of movable finger, **IA** internal angle of chitinized arch, **L** lobe, **LT** lateral tip of chitinized arch, **Msp** patches of microsetae of the male flagellum, **PB** posterior branch of chitinized arch, **Pe** patella ectally, **Pm** patella mesally, **Pmm** mid mesal part of patella, **Pme** mid ectal part of patella, **S** serrula, **Ter** row of tibia externally, **Tmr** row of tibia medially, **Tir** row of tibia internally, **Vm** ventro-median setae of the abdomen and flagellum, **Vl** ventro-lateral setae of the abdomen and flagellum.

## ﻿Taxonomy

### ﻿Family Hubbardiidae Cook, 1899

#### ﻿Genus Bamazomus

Taxon classificationAnimaliaSchizomidaHubbardiidae

Harvey, 1992

7467A1B1-11E1-5EBC-8BAE-9DFEEC1BF8BE

##### Type species.

*Bamazomusbamaga* Harvey, 1992 from Queensland.

##### Comment.

*Bamazomus* resembles *Apozomus* Harvey, 1992 and can be distinguished from it by: 1) spermathecae with numerous lobes vs only with two pairs of lobes; 2) flagellum with posterior process in male vs without posterior process. Currently 11 species of the genus are known to occur from Madagascar to Australia. There is only one anthropochorous species, *B.siamensis* (Hansen, 1905), which is known outside of natural range. It is known from Thailand (type locality), Hawaii, Ryukyu Islands, and Hong Kong. Until now only this species was known from the continental part of Asia, namely from Bangkok (WSC 2024).

#### ﻿ Bamazomus shanghang sp. nov.

Taxon classificationAnimaliaSchizomidaHubbardiidae

80F5C342-A52D-57B8-A7F0-A1CE4553C8E4

https://zoobank.org/E6A3A077-5D27-41CF-95E9-2A4EE4287F38

**Figure 1.**
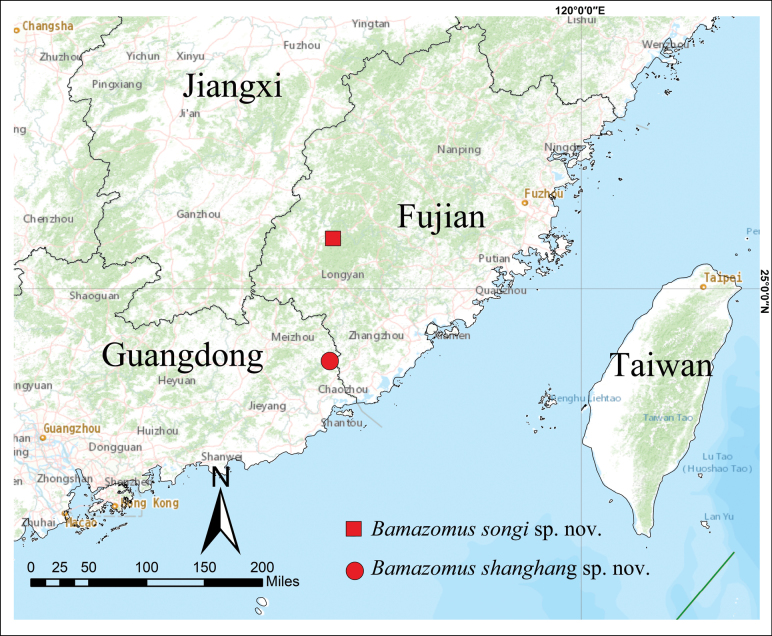
Type localities of *Bamazomussongi* sp. nov. (square) and *B.shanghang* sp. nov. (circle).

**Figure 2.**
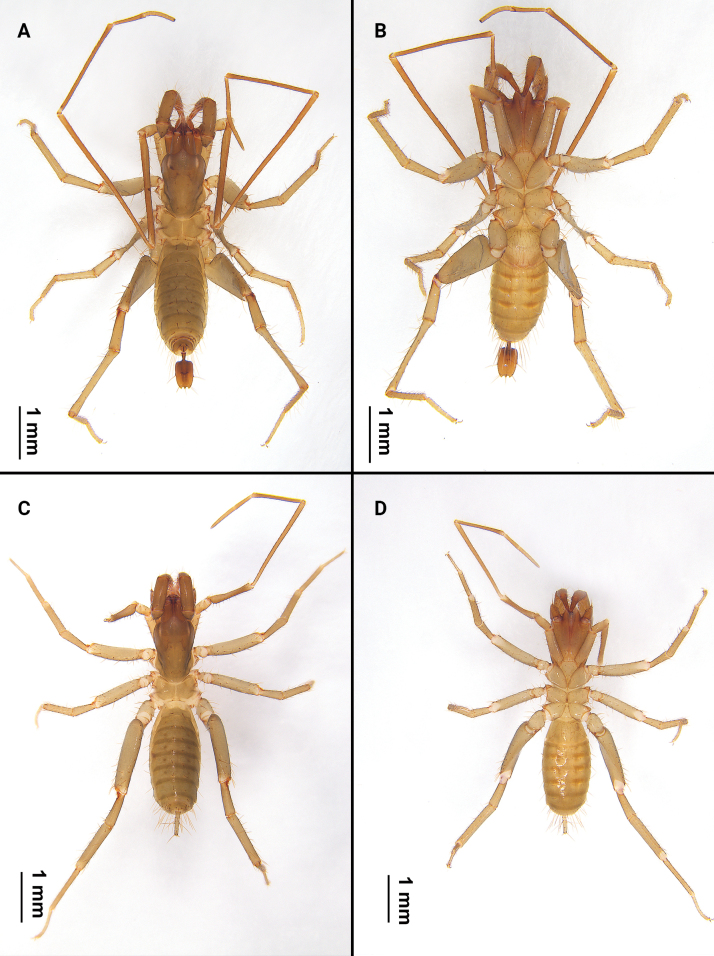
Habitus of *Bamazomusshanghang* sp. nov., holotype male and paratype female **A** male, dorsal view **B** same, ventral view **C** female paratype, dorsal view **D** same, ventral view.

**Figure 3.**
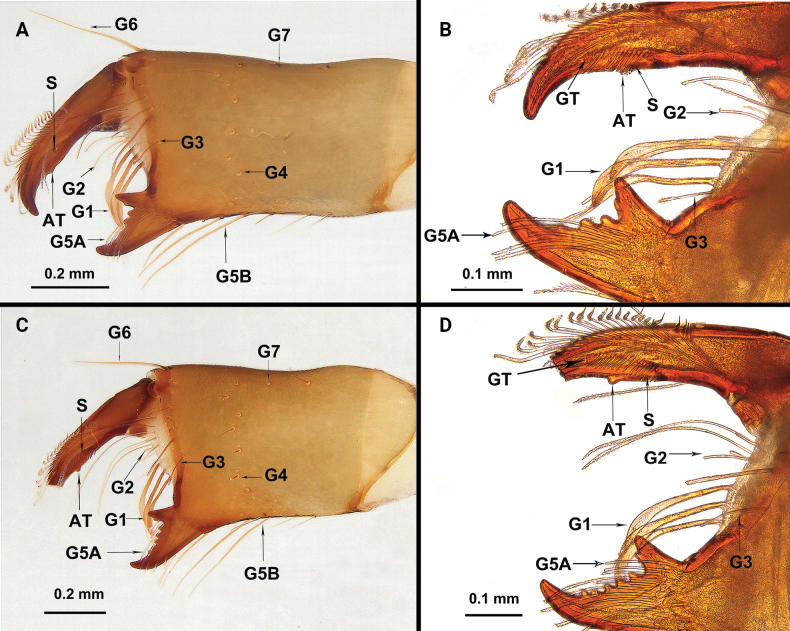
Chelicerae of *Bamazomusshanghang* sp. nov., holotype male and paratype female **A** male, mesal view **B** same, movable finger and fixed finger **C** female (tip broken), mesal view **D** same, movable finger and fixed finger. Abbreviations: AT = accessory tooth of movable finger, G = setal group numbers of chelicerae, GT = guard tooth of movable finger, S = serrula.

**Figure 4.**
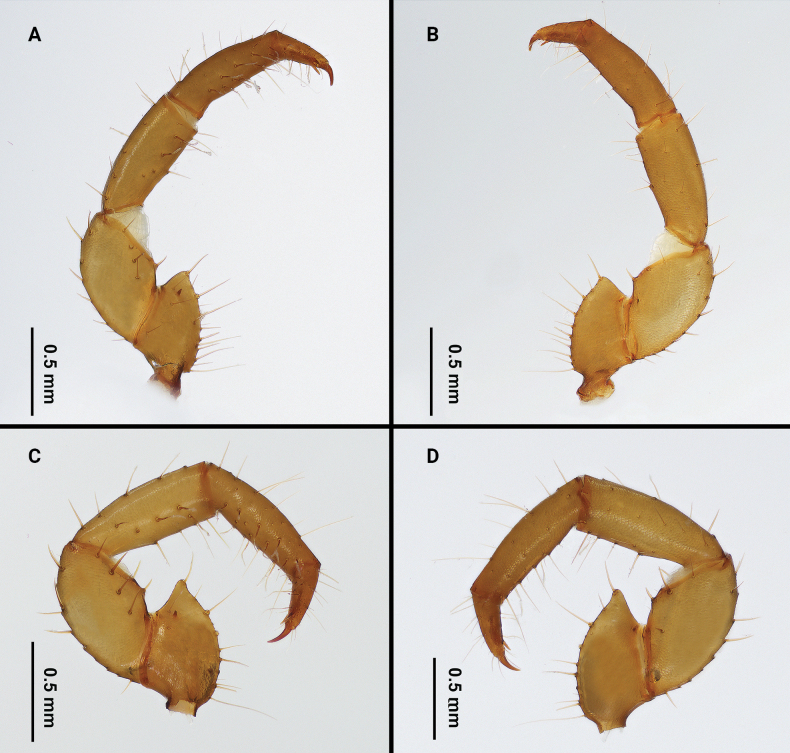
Palps of *Bamazomusshanghang* sp. nov., holotype male and paratype female **A** male, mesal view **B** same, ectal view **C** female, mesal view **D** same, ectal view.

**Figure 5.**
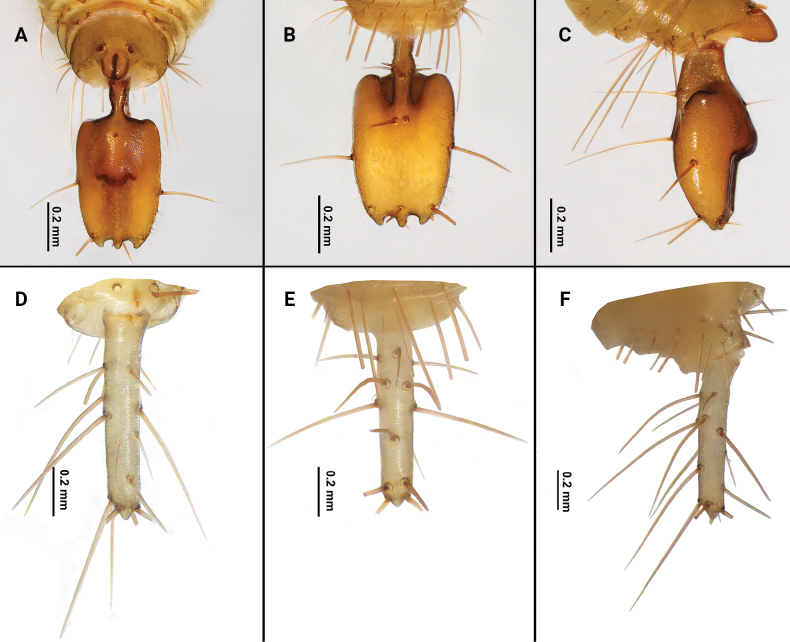
Flagellum of *Bamazomusshanghang* sp. nov., holotype male and paratype female **A** male, dorsal view **B** same, ventral view **C** same, lateral view **D** female, dorsal view **E** same, ventral view **F** same, lateral view.

**Figure 6.**
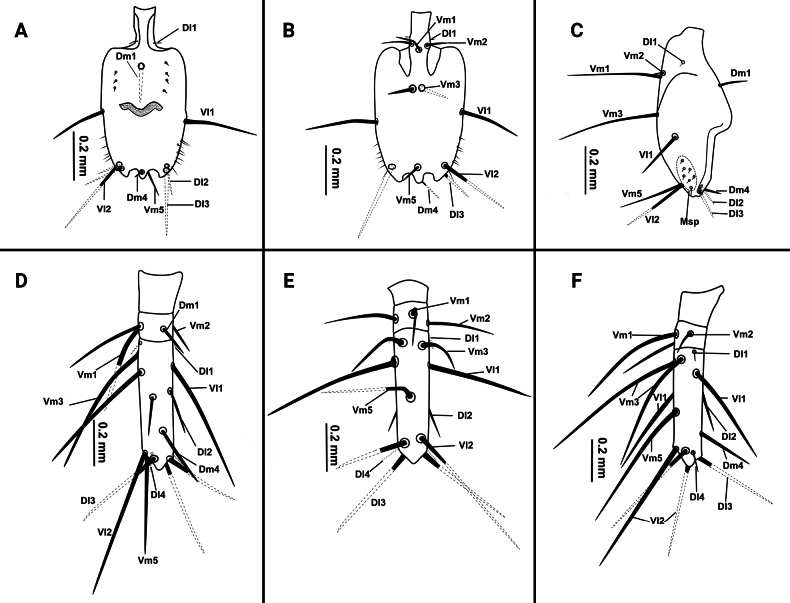
Flagellum of *Bamazomusshanghang* sp. nov., holotype male and paratype female **A** male, dorsal view **B** same, ventral view **C** same, lateral view **D** female, dorsal view **E** same, ventral view **F** same, lateral view. Abbreviations: Dm = dorso-median setae of abdomen and flagellum, Dl = dorso-lateral setae of the abdomen and flagellum, Vm = ventro-median setae of the abdomen and flagellum, Vl = ventro-lateral setae of the abdomen and flagellum.

**Figure 7.**
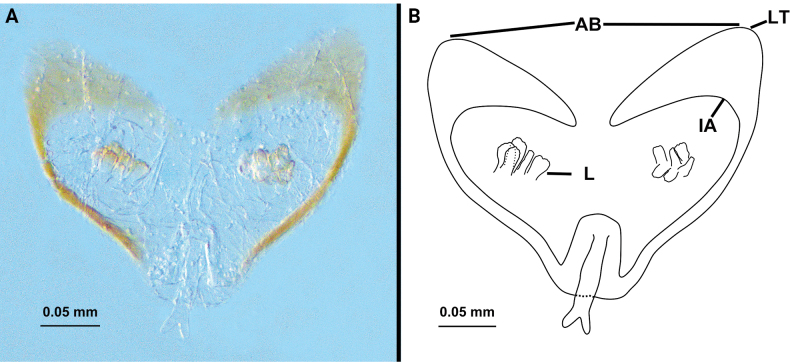
Spermathecae of *Bamazomusshanghang* sp. nov. **A, B** female paratype, dosal view. Abbreviations: AB = anterior branch of chitinized arch, IA = internal angle of chitinized arch, L = lobe, LT = lateral tip of chitinized arch.

Figs 1 , 2 , 3 , 4 , 5 , 6 , 7 , Table 1 上杭巴马加盾

##### Type material.

***Holotype*** ♂ (MHBU-2023312-1), China: Fujian Province, Longyan City, Shanghang County, Shanghang National Forest Park, 25.6364°N, 116.9097°E, 672 m elev., 22.VII.2023, leg. T. Zheng, J.-X. Gong. ***Paratype***: 1♀ (MHBU-2023312-2), same data as the holotype.

##### Etymology.

The specific name is a noun in apposition, referring to the name of the type locality.

##### Diagnosis.

The new species resembles *B.siamensis* in having three posterior processes and a small, conical protuberance on the posterior margin of flagellum in the male, and spermathecal lobes with several apical apophyses (Cokendolpher and Reddell 1986: figs 2–4; Hansen and Soerensen 1905: pl. 5, fig. 2g, h), but it can be distinguished by: 1) the absence of duct openings of spermathecal lobes and presence of the incomplete anterior branch of chitinized arch (Fig. 7A, B) vs duct openings present and anterior branch complete (Cokendolpher and Reddell 1986: figs 2–4); 2) the posterior dorsal process of XII segment of opisthosoma semi-oval, blunt, and short (Figs 2A, 5A) vs conical, acuminate, and long (Hansen and Soerensen 1905: pl. 5, fig. 2h); 3) the presence of Dm4 on flagellum, with two rows of microsetae placed dorsolaterally next to Dm1, right row with four microsetae, left row with three microsetae in the male (Figs 5A–C, 6A–C) vs Dm4 and two row of microsetae are absent (Hansen and Soerensen 1905: pl. 5, fig. 2g, h).

##### Description.

***Holotype* Male** (Fig. 2A, B): measurements as in Table 1. Colour: light brownish. Prosoma: anterior process of propeltidium with three setae (pair of setae followed by a single seta) followed by five pairs of dorsal setae (2+2+2+2+2); eye spots distinct. Mesopeltidia separated. Metapeltidium divided. Anterior sternum with 14 setae (including two sternapophysial setae); posterior sternum triangular with six setae.

**Table 1.** Measurements (mm) of *Bamazomussongi* sp. nov. and *B.shanghang* sp. nov.

		*Bamazomusshanghang* sp. nov.		*Bamazomussongi* sp. nov.	
		Male (Holotype)	Female (Paratype)	Male (Holotype)	Female (Paratype) MH BU-ZT5-2
Total Length		4.37	4.52	5.41	5.51
Propeltidium	Length	1.45	1.53	1.39	1.49
	Width	0.87	0.87	0.97	0.86
Flagellum	Length	0.67	0.78	0.69	0.49
	Width	0.35	0.09	0.34	0.09
	Height	0.30	0.09	0.30	0.09
Leg I	Coxa	1.00	0.81	1.17	0.73
	Trochanter	0.59	0.46	0.79	0.46
	Femur	2.37	1.51	2.48	1.45
	Patella	3.16	2.03	3.50	1.93
	Tibia	2.38	1.47	2.26	1.44
	Basitarsus	0.64	0.43	0.76	0.44
	Telotarsus	1.48	0.65	0.91	0.55
	Total	11.57	7.36	11.87	7.00
Leg IV	Trochanter	0.64	0.53	0.59	0.51
	Femur	1.84	1.59	2.23	1.58
	Patella	0.90	0.68	0.99	0.66
	Tibia	1.48	1.16	1.68	1.10
	Basitarsus	1.24	0.99	1.46	0.99
	Telotarsus	0.81	0.71	0.88	0.70
	Total	7.39	6.14	8.34	5.98
Pedipalp	Trochanter	0.66	0.65	0.37	0.71
	Femur	0.77	0.74	0.85	0.57
	Patella	0.78	0.73	0.83	0.64
	Tibia	0.73	0.71	0.79	0.62
	Tarsus	0.37	0.28	0.34	0.36
	Total	2.97	2.78	3.28	3.46

Chelicerae (Fig. 3A, B): movable finger: serrula with 17 teeth, guard tooth present, with one prominent accessory tooth at subterminal part of movable finger. Fixed finger with two large teeth and four smaller teeth, proximal tooth with one tiny, blunt lateral tooth. Setation: setal group formula: 3–9–5–4–10–6–1–6. G1 with three spatulate setae; G2 composed of nine smooth setae; G3 with five setae, feathered apically and smooth basally; G4 consisting of four small setae, smooth, basally thick, distally elongated; G5A with 10 similarly sized setae, feathered apically and smooth basally, length almost equal to movable finger; G5B with six setae, basal two short and smooth, apical four longer and feathered; G6 with one smooth seta about 3/5 of movable finger length; G7 with seven smooth setae.

Palps (Fig. 4A, B): 2.05 times longer than propeltidium; trochanter with apical process, blunt apical process with angle of about 70°; mesal surface of trochanter with two setae near ventral margin and two setae near dorsal margin; with one small mesal spur. Femur 1.7 times longer than high; ventral margin on ectal surface with acuminate setae Fe1, Fe5, Fev1, Fev2 and one dorsal seta Fed3; mesal surface with row of four ventral setae (Fmv 1–4) and one dorsal seta Fmd3. Patella with three acuminate setae Pe and one seta Pme1 on ventro-ectal surface; with three feathered setae Pm and one seta Pmm3 on ventro-mesal surface. Setae formula on tibia 3–3–5. Tarsal spurs asymmetrical.

Legs: leg I, basitarsal–telotarsal proportions: 32: 5: 6: 6: 7: 6: 12. Femur IV 3.14 times longer than high.

Opisthosoma: tergite I with three pairs of microsetae anteriorly and one pair of Dm; tergite II with three pairs of microsetae anteriorly and pair of Dm; tergites III–VII with one pair of Dm setae each; tergite VIII with pairs Dm and Dl2; tergite IX with pairs Dm, Dl1 and Dl2. Segments X, XI telescoped, with setal pairs Dm, Dl1, Dl2, Vm2, Vl1, Vl2, and single Vm1; segment XII with Dm, Dl1, Dl2, Vm2, Vl1, Vl2, and single Vm1, with posterodorsal process. Sternites II–VII with two irregular rows of setae each; genital plate with scattered setae.

Flagellum (Figs 5A–C, 6A–C): nearly rectangular in shape; 1.94 times longer than wide; posterior margin with three posterior processes; dorsal side with small, conical protuberance; setation: seta Dm1 situate base of bulb, two rows of microseate located dorsolaterally next to Dm1, right row with four microsetae, left row with three microsetae; Dm4 at same level as Dl3; Dl2 anterior to Dm4; both sides of pedicel with Dl1; Vm1 posterior to Vm2; Vm3 anterior to Vl1; Vm5 at same level as Vl2; two Msp between Vl1 and Vl2.

**Female. *Paratype*** (Fig. 2C, D): measurements as in Table 1. Colour: light brownish. Palps (Fig. 4C, D) similar to male, 1.82 times longer than propeltidium, setae formula on tibia 4–2–3. Prosoma: anterior process of propeltidium with three setae (pair of setae followed by single seta) followed by five pairs of dorsal setae. Flagellum (Figs 5D–F, 6D–F) with three flagellomeres, setation: Vl1 anterior to Dl2; Vm1 at same level as Vm2; Dl4 posterior to Dm4; Vm3 anterior to Vl1; Dl3 at same level as Vl2; Dl1 posterior to Vm2; Vm5 posterior to Dl2. Spermathecae (Fig. 7A, B) with five or six pairs of lobes, short and thick, with some apical apophyses. Chitinized arch heart-shaped, with a wide LT and with curved, wide and incomplete anterior AB. Gonopod distal bifurcation. Chelicerae (Fig. 3C, D): movable finger with one prominent accessory tooth; serrula with 17 teeth. Fixed finger with two large teeth and six smaller teeth, proximal tooth with one tiny, blunt lateral tooth. Setal group formula 3–9–5–4–10–6–1–6.

##### Comments.

The female of new species has a more pronounced apical process of the palpal trochanter than the male, which is uncommon and generally opposite to other schizomids. Usually, there are two or three G4 on the chelicerae in Hubbardiidae, and these are concentrated in the lower row. G4 are easily confused with G7, but it is believed that G4 can be distinguished by their short setae which are thickened at the base (vs long setae which are not basally thickened), as seen in the new species.

##### Habitats.

The new species was collected under a heap of leaf-covered stones. The female specimen was collected from the underside of a stone, while the male was found in the ground under the stones.

##### Distribution.

This species is known only from the type locality (Fig. 1).

#### ﻿ Bamazomus songi sp. nov.

Taxon classificationAnimaliaSchizomidaHubbardiidae

5F42EAFB-B551-5A90-9B88-21AF6BFBE47C

https://zoobank.org/DD7C1E05-5C3C-4408-9321-20D5367790B6

**Figure 8.**
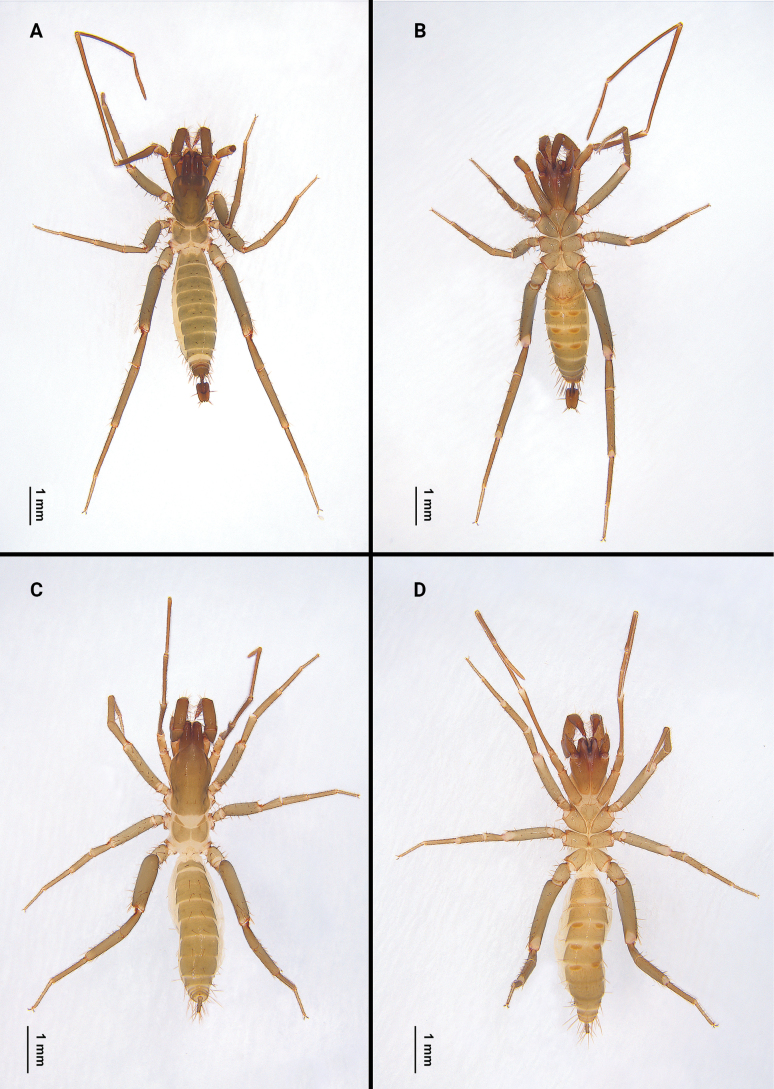
Habitus of *Bamazomussongi* sp. nov., holotype male and paratype female **A** male, dorsal view **B** same, ventral view **C** female paratype, dorsal view **D** same, ventral view.

**Figure 9.**
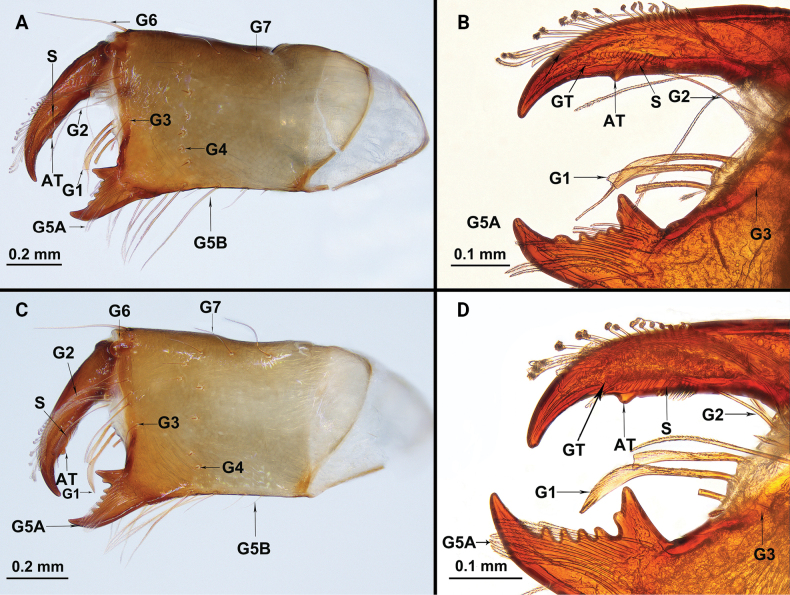
Chelicerae of *Bamazomussongi* sp. nov., holotype male and paratype female **A** male, mesal view **B** same, movable finger and fixed finger **C** female, mesal view **D** same, movable finger and fixed finger. Abbreviations: AT = accessory tooth of movable finger, G = setal group numbers of chelicerae, GT = guard tooth of movable finger, S = serrula.

**Figure 10.**
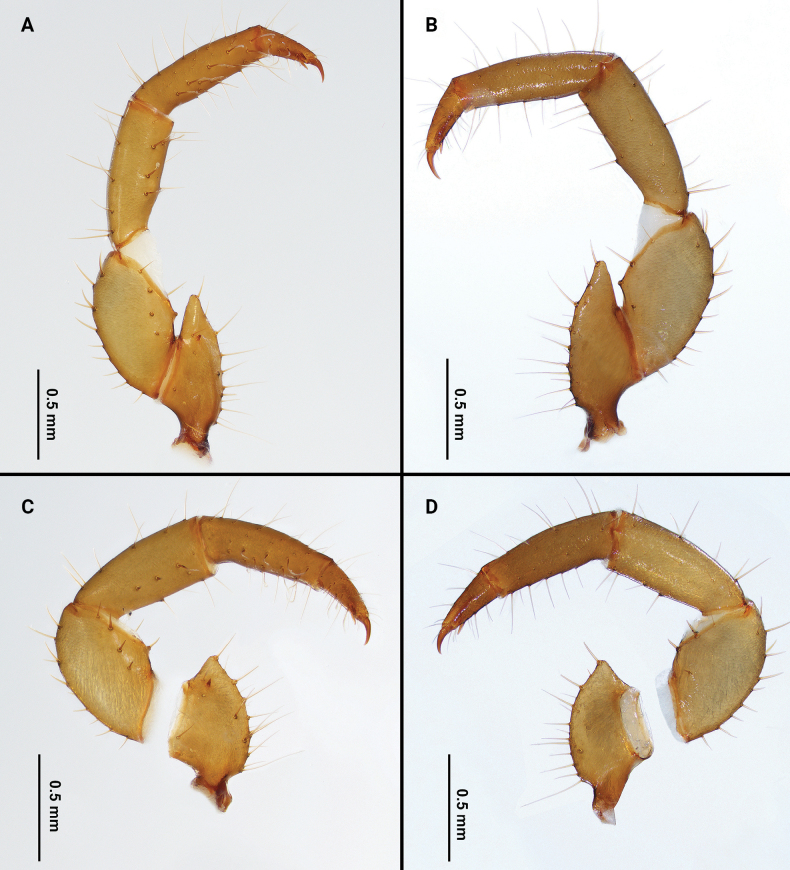
Palps of *Bamazomussongi* sp. nov., holotype male and paratype female **A** male, mesal view **B** same, ectal view **C** female, mesal view **D** same, ectal view.

**Figure 11.**
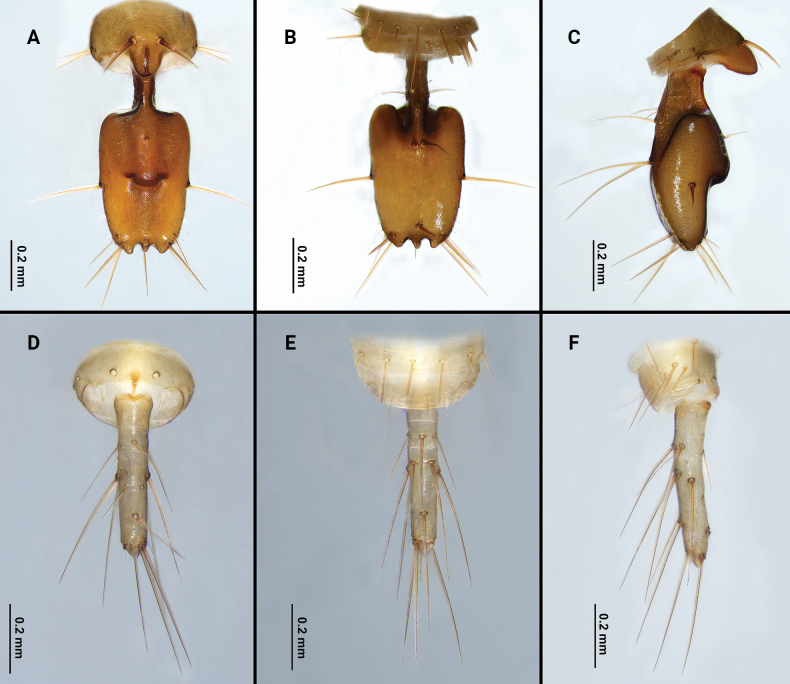
Flagellum of *Bamazomussongi* sp. nov., holotype male and paratype female **A** male, dorsal view **B** same, ventral view **C** same, lateral view **D** female, dorsal view **E** same, ventral view **F** same, lateral view.

**Figure 12.**
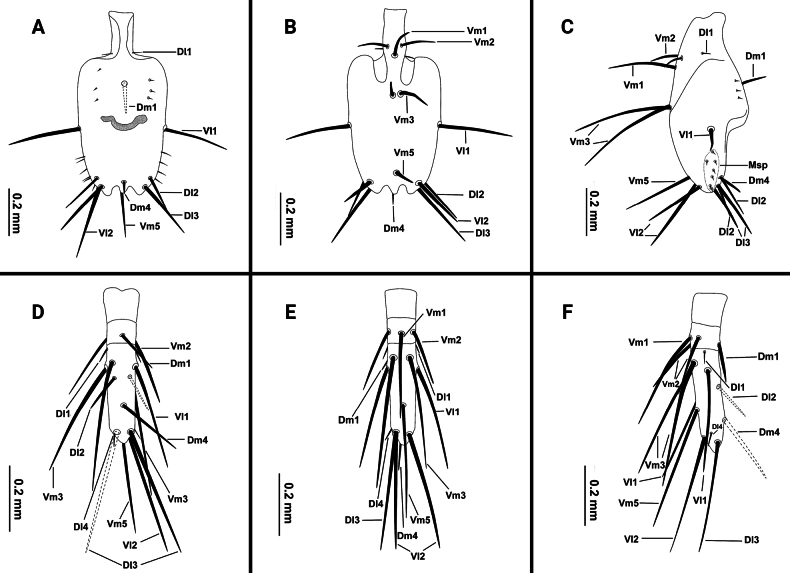
Flagellum of *Bamazomussongi* sp. nov., holotype male and paratype female **A** male, dorsal view **B** same, ventral view **C** same, lateral view **D** female, dorsal view **E** same, ventral view **F** same, lateral view. Abbreviations: Dm = dorso-median setae of abdomen and flagellum, Dl = dorso-lateral setae of the abdomen and flagellum, Vm = ventro-median setae of the abdomen and flagellum, Vl = ventro-lateral setae of the abdomen and flagellum.

**Figure 13.**
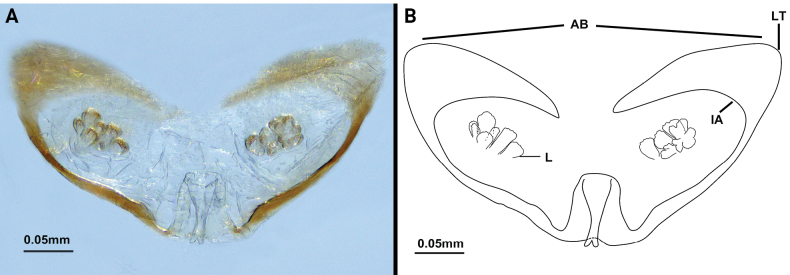
Spermathecae of *Bamazomussongi* sp. nov. **A, B** female paratype, dosal view. Abbreviations: AB = anterior branch of chitinized arch, IA = internal angle of chitinized arch, L = lobe, LT = lateral tip of chitinized arch.

Figs 1 , 8 , 9 , 10 , 11 , 12 , 13 , Table 1 宋氏巴马加盾

##### Type material.

***Holotype*** ♂ (MHBU-ZT5-1), China: Guangdong Province, Chaozhou City, Raoping County, Raoyang Town, Gangxia Village, 24.0924°N, 116.8814°E, 177 m elev., 23.VIII.2023, leg. J.-X. Gong. ***Paratype***: 2♂ 4♀ (MHBU-ZT5-2), same data as the holotype.

##### Etymology.

The specific name is a patronym in honour of the late academician Daxiang Song (1935–2008), a scholar of arachnology who was the first to describe Schizomida from China.

##### Diagnosis.

*Bamazomussongi* sp. nov. resembles *B.shanghang* sp. nov. in having three posterior processes and a small, conical protuberance on the posterior margin of flagellum in the male, and in having spermathecal lobes with several apical apophyses and an incomplete anterior branch (Figs 5A, B, 6A, B, 7A, B), but can be distinguished by: 1) the presence of seven or eight pairs of spermathecal lobes (Fig. 13A, B) vs five or six pairs of lobes (Fig. 7A, B); 2) the short, proximally gonopod broad and wide chitinized arch (Fig. 13A, B) vs the long, proximally narrow gonopod and narrow chitinized arch (Fig. 7A, B); 3) the Dm4 anterior to the middle dorsal process of flagellum (Figs 11A, 12A) vs Dm4 on the middle dorsal process (Figs 5A, 6A); 4) the Dm4 anterior to Dl3 and the Vm5 anterior to Vl2 on flagellum, the long, acuminate trochanter apical process on pedipalps in the male (Figs 10A, B, 11A–C, 12A–C) vs the Dm4 posterior to Dl3, the Vm5 at same level as Vl2, blunt and short (Figs 4A, B, 5A–C, 6A–C).

##### Description.

***Holotype* Male** (Fig. 8A, B): measurements as in Table 1. Colour: brownish. Prosoma: anterior process of propeltidium with three setae (pair of setae followed by single seta) followed four pairs of dorsal setae (2+2+2+2); eye spots distinct. Mesopeltidia separated. Metapeltidium divided. Anterior sternum with 11 setae (including two sternapophysial setae); posterior sternum triangular with six setae.

Chelicerae (Fig. 9A, B): movable finger: serrula with 17 teeth, guard tooth present, with one prominent accessory tooth at subterminal part of movable finger. Fixed finger with two large teeth and four smaller teeth, proximal tooth with one tiny, blunt lateral tooth. Setation: setal group formula: 3–6–4–5–13–10–1–5. G1 with three spatulate setae; G2 composed of six feathered setae; G3 with four setae, feathered apically and smooth basally; G4 consisting of five small setae, smooth, basally thick, distally elongated; G5A with 13 similar sized setae, feathered apically and smooth basally, length almost equal to movable finger; G5B with 10 setae, basal three short and smooth, apical seven longer and feathered; G6 with one smooth seta about 1/2 of movable finger length; G7 with five smooth setae.

Palps (Fig. 10A, B): 2.9 times longer than propeltidium length; trochanter with apical process, pointed apical process with angle of about 45°; mesal surface of trochanter with three setae near ventral margin and one seta near dorsal margin; with one small mesal spur. Femur 2.4 times longer than high; ventral margin on ectal surface with acuminate setae Fe1, Fe5, Fev1, Fev2 and one dorsal setae Fed3; mesal surface with a row of four ventral setae (Fmv 1–4) and one dorsal seta Fmd3. Patella with three acuminate setae Pe and one seta Pme1 on ventro-ectal surface; with three feathered setae Pm and one seta Pmm3 on ventro-mesal surface. Setae formula on tibia 5–3–6. Tarsal spurs asymmetrical.

Legs: leg I, basitarsal–telotarsal proportions: 37: 5: 6: 7: 6: 7: 13. Femur IV 3.70 times longer than high.

Opisthosoma: tergite I with three pairs of microsetae anteriorly and pair of Dm; tergite II with three pairs of microsetae anteriorly and pair of Dm; tergites III–VII with one pair of Dm setae each; tergite VIII with pairs Dm and Dl2; tergite IX with pairs Dm, Dl1, and Dl2. Segments X, XI telescoped, with setal pairs Dl1, Dl2, Vm2, Vl1, Vl2, and single Vm1; segment XII with Dm, Dl1, Dl2, Vm2, Vl1, Vl2, and single Vm1, with posterodorsal process. Sternites II–VII with two irregular rows of setae each; genital plate with scattered setae.

Flagellum (Figs 11A–C, 12A–C): nearly rectangular in shape; 1.75 times longer than wide; posterior margin of flagellum with three posterior processes; the dorsal side with a small, conical protuberance; setation: seta Dm1 situate base of bulb, two rows of microseate placed dorsolaterally next to Dm1, each row with three microsetae; Dm4 anterior to Dl3; Dl2 anterior to Dm4; both sides of pedicel with Dl1; Vm1 posterior to Vm2; Vm3 anterior to Vl1; Vm5 anterior to Vl2; two Msp between Vl1 and Vl2.

**Female. *Paratype*** (Fig. 8C, D): measurements as in Table 1. Colour: brownish. pedipalps (Fig. 10C, D) similar to male, 2.30 times longer than propeltidium length, setae formula on tibia 4–5–4. Prosoma anterior process of propeltidium with three setae (pair of setae followed by single seta) followed by four pairs of dorsal setae. Flagellum (Figs 11D–F, 12D–F) with three flagellomeres. Setation: Vl1 anterior to Dl2; Vm1 at same level as Vm2; Dl4 posterior to Dm4; Vl2 anterior to Dl3; Dl1 anterior to Vm3; Dm4 posterior to Vm5. Spermathecae (Fig. 13A, B) with seven or eight pairs of short, thick lobes; spermathecal lobes with many small apical apophyses and with slight bifurcations. Chitinized arch wide, heart-shaped; with a wide LT and with curved, wide and incomplete AB. Gonopod short and the base broad, with distal bifurcation. Chelicerae (Fig. 9C, D): movable finger with one prominent accessory tooth; serrula with 16 teeth. Fixed finger with two large teeth and five smaller teeth between these, including one tiny, blunt lateral tooth on proximal tooth. Setal group formula 3–6–5–4–11–9–1–5.

##### Comments.

The number of G4 on the chelicerae of both *B.songi* sp. nov. and *B.shanghang* sp. nov. is similar, with all of them exceeding three. Therefore, previous descriptions of some species in the Hubbardiidae might have confused G4 and G7.

##### Habitats.

Specimens of *Bamazomussongi* sp. nov. were collected under leaf-covered, relatively wet stones near a stream.

##### Distribution.

This species is known only from the type locality (Fig. 1).

## Supplementary Material

XML Treatment for Bamazomus

XML Treatment for Bamazomus shanghang

XML Treatment for Bamazomus songi

## ﻿Additional information

### Author ORCIDs

Tao Zheng https://orcid.org/0009-0000-5936-9097

Jiaxian Gong https://orcid.org/0009-0009-4753-0360

Feng Zhang https://orcid.org/0000-0002-3347-1031

### Data availability

All of the data that support the findings of this study are available in the main text.
